# Delivery of Molecules Using Nanoscale Systems for Cancer Treatment and/or Diagnosis

**DOI:** 10.3390/pharmaceutics14040851

**Published:** 2022-04-13

**Authors:** Marina Santiago Franco, Yu Seok Youn

**Affiliations:** 1 Institute of Radiation Medicine (IRM), Department of Radiation Sciences (DRS), Helmholtz Zentrum München, 85764 Neuherberg, Germany; 2 School of Pharmacy, Sungkyunkwan University, 2066 Seobu-ro, Jangan-gu, Suwon 16419, Gyeonggi-do, Korea; ysyoun@skku.edu

Since clinical approval of the first liposomal formulation encapsulating a chemotherapeutic agent, nanoscale delivery systems have been a rapidly developing science. This Special Issue highlights the current advances and challenges in the broad theme of nanosystem development for the delivery of molecules to treat and/or diagnose tumors. A series of 10 original articles and 4 review articles written by researchers working in 11 countries were published. These publications provide the reader with a broad overview of some of the key strategies for nanocarrier design. These strategies are brought together by the wide compositional variety of these systems and the diversity of molecules that may be carried for functionalization strategies, codelivery, and combination with other treatment modalities.

Pinto et al. [1] reported the formulation of nanostructured lipid carriers containing doxorubicin (NLC–DOX). The formulation aims to prevent mucositis, one of the main recurring adverse effects of DOX. In an in vivo experiment, they demonstrated that NLC–DOX could attenuate DOX-induced mucositis in mice compared with DOX-free treatment. The formulation prevented morphological alterations, such as the shortening of crypt depth and villus height. Intestinal permeability was preserved, the expression of tight junctions increased, and inflammatory cytokines associated with mucositis were decreased in mice treated with NLC–DOX.

Bolaños et al. [2] prepared gold nanorods and gold nanoprisms coated with albumin functionalized with the cell-penetrating peptide octaarginine. When evaluated in vitro, improved internalization was observed for both nanoparticles coated with octaarginine-functionalized albumin compared to non-functionalized ones. These results validated the novel nanoconstructs as potential candidates for biomedical applications with improved biocompatibility and internalization.

López-Barrera et al. [3] developed chitosan-carrying-glutathione nanoparticles in combination with DOX. Glutathione, one of the primary endogenous antioxidants, is associated with redox state regulation. Therefore, the strategy aimed at reducing the oxidative stress induced by DOX, a key factor in its high toxicity. In vitro evaluation in two breast cancer cell lines suggested that the formulation reduces the oxidative stress induced by DOX interacting with reactive oxygen species, free radicals, or antioxidant enzymes. Additionally, they reported that the formulation increased caspase-3 activity, leading to apoptosis and impaired cell proliferation by decreasing Ki67 levels.

Huang et al. [4] designed hyaluronic-acid-based PEGylated nanoparticles to codeliver epigallocatechin-3-gallate (EGCG) and DOX. These nanoparticles were conjugated to fucoidan and D-alpha-tocopherylpoly(ethylene glycol) succinate for targeting P-selectin- and CD44-expressing gastric tumors. The authors demonstrated in vitro that the nanoparticles containing EGCG/DOX led to a better synergistic antiproliferation effect compared with the free combination of EGCG/DOX. This reflected a higher antitumor activity for the formulation in the orthotopic gastric tumor mouse model.

Vindigni et al. [5] designed aptamer-functionalized nanocages. The aptamer of choice was AS1411, which recognizes and binds specifically and with high affinity to nucleolin (a protein overexpressed on the surface of many tumor cells), inhibiting cell proliferation and inducing cell death. To enhance AS1411 stability and therapeutic efficacy, it was used to functionalize octahedral DNA nanocages. The aptamer acts as a selective tumor-targeting ligand when linked to the nanocages. The formulation presented high stability in serum and rapid and selective internalization in cancer cells. The anticancer activity was reported to be increased by over 200-fold compared with the free aptamer. Different intracellular trafficking for the formulation compared with the free aptamer was also reported.

Yang et al. [6] explored the ability of mesoporous silica nanoparticles (MSNs) to absorb biomaterials, such as antigens. They developed MSNs functionalized with (3-aminopropyl) triethoxysilane and investigated the potential of this formulation to deliver antigens to the tumor, enhancing the abscopal effect after radiotherapy. In vivo experiments in mice with bilateral hepatocellular carcinoma showed promising results, with a significant volume reduction in the non-irradiated tumor when radiotherapy+MSNs were combined in the primary tumor.

Krivitsky et al. [7] developed a sulfonated amphiphilic poly(α)glutamate nanocarrier as a delivery vehicle for siRNA targeting Plk1 genes to chemo-resistant glioblastoma tumors. The brain targeting ability of this formulation was improved by decorating it with sulfonate groups. These groups have a selective affinity towards P-selectin, a transmembrane glycoprotein overexpressed in glioblastoma. In vitro studies showed the potential of the formulation to be internalized by glioblastoma cells, leading to specific gene silencing and affecting its proliferation.

Cho et al. [8] prepared a nanostructured DOX prodrug consisting of an albumin-binding maleimide group, cathepsin-B-cleavable peptide (Phe-Arg-Arg-Gly), and DOX, designed to be activated in cathepsin-B-overexpressed tumor cells. It was demonstrated in vivo using breast tumor mice models that the formulation enhanced DOX accumulation in the tumor by passive targeting. It also induced potent antitumor efficacy due to the selective release of DOX from the formulation in the tumor cells. Furthermore, lower toxicity was observed towards normal tissues due to their low expression of cathepsin B, guaranteeing the formulation’s safety.

Cohen et al. [9] developed PEGylated nanostructured lipid carriers (NLCs) decorated with a targeting ligand (Glu-Urea-Lys) to the prostate-specific membrane antigen. These NLCs were loaded with the anti-microtubule agent cabazitaxel. In their article, an extensive characterization of this formulation was presented, as well as its selective targeting, internalization, and growth-inhibitory activity in vitro against prostate cancer cells.

Lee et al. [10] formulated an in situ injectable multifunctional PEG hydrogel system to provide photodynamic, photothermal, and chemotherapeutic effects to hypoxic tumors. This system was prepared with (i) paclitaxel-bound albumin nanoparticles with chlorin-e6 (a photosensitizer)-conjugated bovine serum albumin and indocyanine green (a deep-penetrating NIR dye) and (ii) an albumin-stabilized perfluorocarbon nano-emulsion (to provide oxygen) in a PEG hydrogel. Under laser irradiation, the hydrogel induced hyperthermia as well as photodynamic cell death, while ultrasound irradiation triggered the release of oxygen. This allowed for the significantly enhanced killing of murine breast cancer cell spheroids in vitro, as well as suppressed tumor growth in 4T1 cell-xenograft mice.

Sizikov et al. [11] reviewed magnetofection, a delivery method for nucleic acids carried in magnetic nanoparticles under the guidance of a magnetic field. A special focus was given to the structure and coating of the magnetic nanocarriers developed so far. Possible ways to improve the technology, its challenges, and prospects were also discussed.

Yang et al. [12] provided a critical overview of the synergistic enhancement of photodynamic therapy (PDT) by combining it with the codelivery of molecules in nanocarriers. In this extensive review, nanocarriers used to codeliver combinations of different molecules, such as photosensitizers, photothermal agents, hyperthermia agents, scintillators/radionuclides, sonophotosensitizers, and chemotherapeutic agents, are reported.

On the other hand, the use of nanocarrier-assisted delivery in combination with PDT was the focus of the review article by Carobeli et al. [13]. Based on an extensive compilation of in vitro and in vivo data, this review highlights the potential of formulations of phthalocyanines as photosensitizers for different types of cervical cancer.

Finally, Gomes and Franco [14] reviewed the combination of nanocarrier-assisted molecule delivery and radiotherapy. Formulations designed to encapsulate molecules that improve the effects of radiotherapy on tumor cells, modulate the tumor microenvironment by relieving hypoxia, or boost the abscopal effect are described. The employment of radiotherapy to trigger molecule delivery from nanocarriers and the use of minibeam or microbeam radiotherapy as possibilities to modulate the enhanced permeability and retention (EPR) effect were also covered in this review.

Overall, the articles in this Special Issue highlight a very active field, and we expect to see an increasing number of nanocarriers reaching cancer clinical trials.
